# Nonrandom segregation of sister chromosomes by *Escherichia coli* MukBEF

**DOI:** 10.1073/pnas.2022078118

**Published:** 2021-08-12

**Authors:** Jarno Mäkelä, Stephan Uphoff, David J. Sherratt

**Affiliations:** ^a^Department of Biochemistry, University of Oxford, Oxford OX1 3QU, United Kingdom

**Keywords:** MukBEF, MatP, SMC, chromosome organization, DNA replication

## Abstract

Structural maintenance of chromosomes (SMC) complexes contribute to chromosome organization in all domains of life. In *Escherichia coli*, MukBEF, the functional SMC homolog, promotes spatiotemporal chromosome organization and faithful chromosome segregation. Here, we address the relative contributions of MukBEF and the replication terminus (*ter*) binding protein, MatP, to chromosome organization–segregation. We show that MukBEF, but not MatP, is required for the normal localization of the origin of replication to midcell and for the establishment of translational symmetry between newly replicated sister chromosomes. Overall, chromosome orientation is normally maintained through division from one generation to the next. Analysis of loci flanking the replication termination region (*ter*), which demark the ends of the linearly organized portion of the nucleoid, demonstrates that MatP is required for maintenance of chromosome orientation. We show that DNA-bound β_2_-processivity clamps, which mark the lagging strands at DNA replication forks, localize to the cell center, independent of replisome location but dependent on MukBEF action, and consistent with translational symmetry of sister chromosomes. Finally, we directly show that the older (“immortal”) template DNA strand, propagated from previous generations, is preferentially inherited by the cell forming at the old pole, dependent on MukBEF and MatP. The work further implicates MukBEF and MatP as central players in chromosome organization, segregation, and nonrandom inheritance of genetic material and suggests a general framework for understanding how chromosome conformation and dynamics shape subcellular organization.

## Significance

Circular chromosomes in rod-shaped bacteria exist inside a cell in two distinct configurations, “transverse” and “longitudinal,” relative to the long cell axis, with chromosomal loci occupying specific cellular locations in both cases. Bacteria with longitudinal chromosome organization (e.g., *Caulobacter crescentus*) typically tether their origins of replication to the cell membrane and do not undergo overlapping rounds of replication. In contrast, bacteria with transverse organization (e.g., *Escherichia coli*) orient their chromosomes by an unknown mechanism and have lifestyles compatible with overlapping rounds of replication. Here, we address the relative roles of two major players in chromosome organization–segregation and propose a model of how *E. coli* maintains chromosome conformation and orientation inside cells and how this organization is propagated over generations.

Faithful chromosome propagation and inheritance underpin all replicative life. Organisms have evolved a vast range of mechanisms to ensure timely replication and segregation of genetic material. Despite this diversity, highly conserved structural maintenance of chromosomes (SMC) complexes play a central role in the organization of chromosomes in all domains of life. Eukaryotic cells orchestrate replication and segregation in discrete stages, in which newly replicated sister chromosomes are first individualized by condensin and held together by cohesin before being pulled apart by the action of the mitotic spindle and cleavage of cohesin (reviewed in ref. 1). In contrast, in prokaryotes, chromosome replication and segregation are generally not temporally separated and occur progressively (2). Because divergent species have evolved different solutions to the same problem, understanding the contributions of different mechanisms and physical constraints underlying robust chromosome segregation remains a challenge (3–5).

Genetic studies have identified two major classes of proteins implicated in chromosome segregation in bacteria. First, SMC complexes, MukBEF, MksBEF, and Smc-ScpAB, were initially identified in a screen for *Escherichia coli* mutants that generated anucleate cells as a consequence of a failure to segregate newly replicated chromosomes to daughter cells (6, 7). Second, studies of low–copy plasmid stability identified ParAB*S* systems, which subsequently were shown to have roles in chromosome segregation in many organisms (5). While many bacteria encode one or both of these systems, some, for example *Pseudomonas aeruginosa*, encode two different SMCs and a ParAB*S* system (8, 9). Nevertheless, the deletion of SMC or ParAB proteins has frequently modest if any consequences for chromosome segregation. Consistent with this, it has been proposed that large bacterial chromosomes can utilize repelling entropic effects to facilitate the separation of chromosomes (10), unlike much smaller low–copy number plasmids that require a functional ParAB*S* system for faithful segregation (5). Whatever roles entropic forces may play, studies in diverse bacterial species have demonstrated that chromosomal loci are not positioned randomly in cells (9, 11–15) and that in *E. coli*, MukBEF complexes play an important role in the correct positioning of replication origins and other loci by forming an axial core to the chromosome (16, 17). Continuous axial cores were the most easily visualized in cells in which MukBEF occupancy on the chromosome was modestly increased, while cells with wild-type (WT) MukBEF abundance on chromosomes exhibited more granular structures (17). The axial cores are linear (as opposed to circular) because *matS*-bound MatP displaces MukBEF from the 800-kb *ter* region (17, 18). The absence of MukBEF leads to the formation of anucleate cells during growth and loss of viability at temperatures higher than 22 °C in rich media (16, 19).

In newborn *E. coli* cells with nonoverlapping replication cycles, the origins of replication (*oriC*) are positioned close to the cell center, and the left and right chromosome arms are linearly organized in separate cell halves. Chromosome replication–segregation leads to generation of daughter cells with a chromosome organization identical to their mother cell. Most cells adopt a *left*-*oriC*-*right*-*left*-*oriC*-*right* (*L*-*R*-*L*-*R*) translational symmetry prior to division (12), which requires that either the leading or lagging strand templates are symmetrically segregated to the cell poles (11, 20). In agreement, an elegant chromosome degradation experiment showed that the leading strand templates are segregated toward the cell poles in most cells (21). In theory, cells could also additionally control the fate of the old template strand by nonrandom segregation, designating the destination for each template strand. Coined as “immortal” strand retention, it was originally proposed as a strategy to maintain DNA purity in stem cells while the copied strands, potentially carrying mutations from replication, were segregated to nonstem cell progeny (22). Whether this strategy is actually utilized by stem cells remains controversial (23–25). Immortal strand segregation has been tested in *Caulobacter crescentus* (26, 27) and *Bacillus subtilis* (28); however, none of these studies showed any segregational strand preference between daughter cells.

We lack a mechanistic understanding of how chromosome conformation and orientation is maintained inside a bacterial cell. It also remains unknown how progressive chromosome segregation facilitates nonrandom sister chromosome inheritance in an otherwise apparently symmetrical organism. Here, we address these questions in *E. coli* utilizing microfluidics culturing devices, combined with time-lapse imaging, high-throughput microscopy, and quantitative analysis. We first demonstrate that, in the absence of MukBEF, anucleate cells arise predominantly from the mother cell’s new pole as a consequence of the failure to segregate newly replicated origins in a timely fashion. We show that nascent lagging strands and their templates are directed toward cell centers, a process that is required for the observed translational *L*-*R*-*L*-*R* segregational symmetry and which is perturbed in the absence of MukBEF. Furthermore, we show directly that the older template DNA strand, inherited from previous generations, is preferentially segregated to the old cell pole, dependent on both MukBEF and MatP. Lack of MatP does not perturb translational *L*-*R*-*L*-*R* symmetry; rather, it leads to flipping of chromosome orientation along the longitudinal cell axis, consistent with the observed loss of the older template strand retention at old poles. Taken together, the results provide a model of how MukBEF and its MatP-driven depletion from the *ter* region lead to asymmetric strand and chromosome segregation. The possible functional and evolutionary consequences of this are discussed.

## Results

### In the Absence of MukBEF, Anucleate Cells Arise from the Newer Mother Cell Pole.

**Fig. 1.**
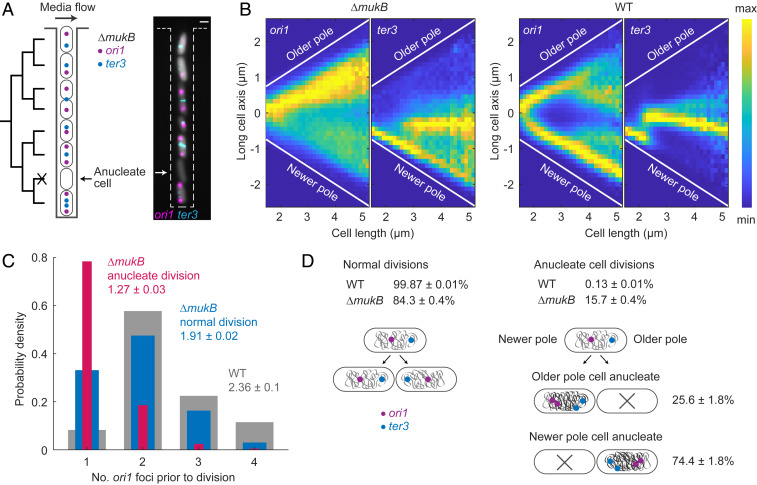
Anucleate cell formation in the absence of MukB is biased toward mother cell newer poles (*A*) Schematic of mother machine microfluidics device and representative cells in a channel. *ΔmukB* cells contain *ori1* and *ter3* FROS markers and a segmentation marker (gray). A nongrowing anucleate cell lacking FROS markers is indicated. (Scale bar, 1 μm.) (*B*) *ori1* and *ter3* localization as a function of cell length in *ΔmukB* (221,057 cells) and WT cells (287,900 cells). Sample numbers with different cell lengths were normalized by the maximum value in each vertical bin. (*C*) Number of *ori1* foci prior to anucleate (one of the daughters is anucleate; 2,444 cells) and normal cell division in *ΔmukB* cells (10,468 cells) and in WT cells (22,224 cells). A two-sample *t* test was used between mean *ori1* numbers prior to anucleate and normal division in *ΔmukB* (*P* value < 10^−5^) and between normal division in *ΔmukB* and WT (*P* value 0.0037). (*D*) Percentage of anucleate cell divisions in *ΔmukB* (14,392 divisions) and WT (22,511 divisions) and the percentage of anucleate cells forming at a mother cell’s old and newer poles in *ΔmukB* (2,269 divisions). Data are from three repeats in *ΔmukB* and two repeats in WT in all analyses.

To understand how anucleate *E. coli* cells form in the absence of MukBEF, we followed the successive cell cycles of *ΔmukB* cells with *oriC* and *ter* (*ori1* and *ter3*, respectively) regions fluorescently labeled by fluorescence repressor–operator system (FROS) markers. A “mother machine” microfluidics device (29, 30) allowed us to follow thousands of cell generations and identify changes in chromosome organization that correlate with chromosome missegregation (Fig. 1*A* and *SI Appendix*, Fig. S1 and Movie S1). Under the growth conditions used (M9 medium supplemented with glucose and essential amino acids at 37 °C), 15.7 ± 0.4% (±SD) of *ΔmukB* cell divisions led to the formation of an anucleate daughter cell, in comparison to 0.13 ± 0.01% (±SD) of WT cell divisions.

In *ΔmukB* cells, *ori1* loci localized preferentially toward the old cell pole (Fig. 1*B*) (16), with the newly replicated sister *ori1* loci frequently remaining in close proximity. Note that replication initiation is not significantly delayed in *ΔmukB* cells as compared to WT cells, in which sister *ori1* separation occurs in a timely manner (18). Meanwhile, *ter3* migration from the newborn cell pole to midcell was only modestly delayed in comparison to WT cells (Fig. 1*B*). Around ∼80% of anucleate cells were generated when duplicated *ori1* loci in mother cells remained together in the old pole cell half prior to cell division (Fig. 1*C*). In contrast, in ∼70% of mother cells, in which chromosome segregation was faithful, *ori1* loci were visible as separate foci. In the ∼30% of *ΔmukB* cells (as compared to ∼10% of WT cells) that had a single *ori1* focus prior to division, which divided and segregated their chromosomes successfully, that single focus must have contained two unsegregated *ori1* loci. This shows that *ori1* numbers are undercounted in our experiments, but it does not change the fact that cells undergoing anucleate division have significantly less separated *ori1* loci.

In anucleate *ΔmukB* cell divisions, daughter cells that inherited two chromosomes divided normally after a modest increase in generation time (normal divisions 63 ± 5 min, anucleate sisters 72 ± 4 min [±SD]; two-sample *t* test *P* value 0.2176; *SI Appendix*, Fig. S1*A*). However, the probability of these cells forming an anucleate cell in subsequent division was 9.1 ± 2% (±SD), significantly lower than for cells born with a single chromosome. During anucleate cell formation, mother cells divided nearly symmetrically (anucleate cell 2.1 ± 0.2 μm and sister cell 2.4 ± 0.2 μm [±SD]; two-sample *t* test *P* value 0.17), with the divisome being placed close to midcell. While the average anucleate cell length at birth did not significantly differ from that of the growing sister (*SI Appendix*, Fig. S1*B*), the bias for the longer growing sister increased with mother cell division size. Note that WT cells had a similar generation time (59 ± 1 min [±SD]) to the *ΔmukB* cells, indicating that the cell-cycle parameters of *ΔmukB* and WT cells are likely to be similar, as reported previously for cells growing in minimal glycerol medium at 30 °C (18).

Finally, we showed that anucleate cells form preferentially at the newer mother cell pole (74.4 ± 1.8% [±SD]; Fig. 1*D*). Therefore, anucleate cell formation is associated with the nucleoid being preferentially retained at the old pole of *ΔmukB* mother cells, while in the case of WT cells the nucleoid is localized closer to the newer pole of a dividing cell (31). We conclude that the mislocalization of *ori1* loci toward the old pole and delayed segregation of newly replicated *ori1* loci are linked to the formation of anucleate cells to the mother cell’s new pole.

### MukBEF and MatP Have Distinct Roles in Generation and Propagation of *left*-*oriC*-*right* Chromosome Organization Over Generations.

**Fig. 2.**
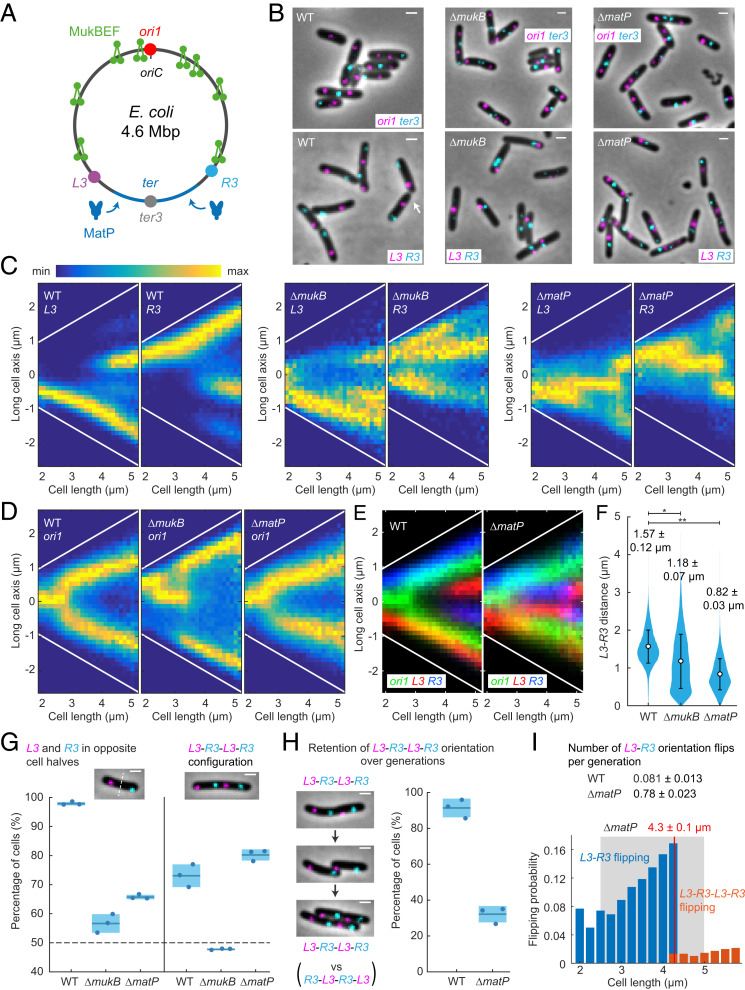
MukBEF and MatP action generates and propagates *L*-*R *chromosome organization in *E. coli*. (*A*) *E. coli* chromosome circular map with *ori1*, *ter3*, *L3*, and *R3* loci. MukBEF complexes are displaced from the 800-kbp *ter* region by *matS* bound MatP. (*B*) Representative images of WT, *ΔmukB*, and *ΔmatP* cells with *ori1* and *ter3* or *L3* and *R3* FROS markers. Note an atypical *R3*-*L3*-*L3*-*R3* configuration in WT (white arrow) in comparison to the standard *L3*-*R3*-*L3*-*R3*. (Scale bars, 1 μm.) *L3* and *R3* localizations (*C*) and *ori1* localizations (*D*) along the long cell axis as a function of cell length in WT (*L3*-*R3* 57,509 cells and *ori1* 42,612 cells), *ΔmukB* (*L3*-*R3* 27,984 cells and *ori1* 54,820 cells), and *ΔmatP* (*L3*-*R3* 46,679 cells and *ori1* 51,350 cells). Sample numbers with different cell lengths were normalized by the maximum value in each vertical bin. Cells are oriented to place *L3* more toward the negative pole (toward figure bottom) or, in the *ori1* data, *ter3* is oriented more toward the negative pole (*SI Appendix*, Fig. S2). White lines denote cell borders. (*E*) Overlay of *ori1* and *L3*-*R3* localization data in WT and *ΔmatP* from *C* and *D*. (*F*) Distance between *L3* and *R3* markers in WT (47,376 cells), *ΔmukB* (15,615 cells), and *ΔmatP* (41,625 cells) in single *L3* and *R3* focus cells. Mean and dispersion (SD) between experiments are shown above each distribution. Error bars denote SD of the cell population. * and ** denote two-sample *t* test of *L3*-*R3* distances between WT and *ΔmukB* (*P* value 0.0081) and WT and *ΔmatP* (*P* value 5 × 10^−4^), respectively. (*G*, *Left*) Percentage of cells with *L3* and *R3* in opposite cell halves in single *L3* and *R3* focus cells (WT 47,376 cells, *ΔmukB* 15,615 cells, and *ΔmatP* 41,625 cells). (*Right*) Percentage of cells with *L3*-*R3*-*L3*-*R3* (or *R3*-*L3*-*R3*-*L3*) configuration (versus *L3*-*R3*-*R3*-*L3* or *R3*-*L3*-*L3*-*R3*) in double *L3* and *R3* focus cells (WT 10,352 cells, *ΔmukB* 2,535 cells, and *ΔmatP* 6,297 cells). The dashed horizontal line indicates random localization, assuming that each sister cell inherits a complete chromosome. (Scale bars, 1 μm.) (*H*) Percentage of cells retaining *L3*-*R3*-*L3*-*R3* orientation (versus flipping to *R3*-*L3*-*R3*-*L3*) from a mother cell to a daughter cell in WT (859 pairs) and *ΔmatP* (1,054 pairs). (Scale bars, 1 μm.) (*I*, *Top*) Number of *L3*-*R3* flipping events (±SD) to *R3*-*L3* (or vice versa) during a cell cycle in WT (3,059 cells) and *ΔmatP* cells (4,102 cells) (*SI Appendix*, Fig. S2 *I* and *J*). (*Bottom*) Probability of *L3*-*R3* flipping to *R3*-*L3* (or vice versa) (blue) and *L3*-*R3*-*L3*-*R3* flipping to *R3*-*L3*-*R3*-*L3* (or vice versa) (orange) as a function of cell length in *ΔmatP* (10,362 cells). The flipping probability was normalized by the number of cells in each bin. The gray box indicates the replication period as a function of cell size from Fig. 3. The red vertical line indicates the average cell length at locus duplication (±SD between experiments). Data are from three repeats in all analyses.

Next, we explored the contributions of MukBEF and MatP in dictating *left*-*oriC*-*right* (*L*-*R*) chromosome organization in *E. coli* and in the propagation of these patterns over generations (12, 17). We used strains that allowed us to test the requirements for left and right chromosome arm organization in relation to *oriC* and *ter* in WT, *ΔmatP*, and *ΔmukB* cells (Fig. 2). The left and right chromosome arms were labeled at *L3* and *R3* (−128° and 122° from *oriC*, respectively) with FROS markers, as were *ori1* and *ter3* loci (Fig. 2 *A* and *B*). We used M9 medium supplemented by glycerol and required amino acids at 30 °C to avoid overlapping replication; under these conditions, replication is initiated several minutes after birth and completed before cell division (18). These growth conditions were used for all experiments described in the following, unless otherwise stated.

Newborn WT cells exhibited the distinctive *left*-*oriC*-*right* (*L3*-*R3*) chromosome organization (Fig. 2 *C*–*E*), in which *oriC* remained at the cell center and the chromosome arms (assayed by *L3* and *R3* localization) resided in opposite cell halves (97.8 ± 0.6% [±SD]; Fig. 2 *F* and *G*) (12, 32). During replication–segregation, the pattern was extended into a translationally symmetric *left*-*oriC*-*right*-*left*-*oriC*-*right* (*L3*-*R3*-*L3*-*R3* or *R3*-*L3*-*R3*-*L3*) pattern in 73.1 ± 3.9% (±SD) of WT cells (Fig. 2*G*), compared to mirror symmetric *L3*-*R3*-*R3*-*L3* or *R3*-*L3*-*L3*-*R3* patterns.

In the absence of MukB, the localization of *ori1*, *L3*, and *R3* chromosomal markers was less precise (Fig. 2 *C–E*), with a wide distribution of *L3*-*R3* distances (Fig. 2*F*), fewer *L3* and *R3* foci localizing in opposite cell halves (56.6 ± 3.2% [±SD]; Fig. 2*G*), and a random chance of observing the *L3*-*R3*-*L3*-*R3*/*R3*-*L3*-*R3*-*L3* organization (47.7± 0.2% [±SD]). Note that to obtain a probability less than 50%, cells would have to actively prevent the two chromosomes from having the same orientation. *ter3* migration pattern of *ΔmukB* cells showed a similar localization pattern to WT with even earlier migration to the cell center (*SI Appendix*, Fig. S2*A*), in contrast to the richer medium condition in Fig. 1. Our observations show that the absence of MukBEF causes the impairment of both the distinctive *L*-*R* chromosome organization prior to replication and the *L*-*R*-*L*-*R* organization after replication.

Meanwhile, *ΔmatP* cells exhibited chromosome locus localization patterns strikingly different from that of WT and *ΔmukB* cells (Fig. 2 *C–E*). The average distance between *L3* and *R3* was reduced twofold (Fig. 2*F*), which also prevented *L3* and *R3* from being directed into opposite cell halves (65.7 ± 0.8% [±SD]; Fig. 2*G*). Concomitantly, it also led to the *L3* and *R3* loci being preferentially localized closer to the cell center than in WT cells, where *L3* and *R3* localize toward the cell poles (Fig. 2*E*). The *ter3* pattern was less precise, lacked the stepwise migration pattern to cell center, and exhibited earlier segregation of the locus (*SI Appendix*, Fig. S2*A*), in agreement with previous studies (18). Despite these substantial perturbations, the *L3*-*R3*-*L3*-*R3* organization was retained in *ΔmatP* cells prior to cell division (80.2 ± 1.9% [±SD]), indicating that other processes must act in determining the observed organization.

To determine if the absence of MatP influences chromosome organization–segregation over generations, we followed WT and *ΔmatP* cells using time-lapse imaging. We observed that *ΔmatP* cells retained the *L3*-*R3*-*L3*-*R3* (or *R3*-*L3*-*R3*-*L3*) orientation in only 32.2 ± 4.6% (±SD) of daughter cells, while most WT cells retained the orientation (91.4 ± 5.2% [±SD]; Fig. 2*H* and *SI Appendix*, Fig. S2 *B–E* and Movies S2 and S3). We next assessed when the marker flipping occurs during the cell cycle. Prior to the duplication of *L3* and *R3* loci, *ΔmatP* cells flipped the orientation on average 0.78 ± 0.02 (±SD) times per cell cycle, compared to 0.08 ± 0.01 (±SD) of WT cells (Fig. 2*I* and *SI Appendix*, Fig. S2 *I* and *J*), while the propensity to flip orientation increased with replication–segregation progression, reaching twofold just before the duplication of the *L3* and *R3* loci (Fig. 2*I*; for WT see *SI Appendix*, Fig. S2*K*). Therefore, locus flipping is not restricted to nonreplicating chromosomes. Once duplicated, the *L3*-*R3*-*L3*-*R3* orientation (or *R3*-*L3*-*R3*-*L3*) was found to be stable until cell division in both WT and *ΔmatP* cells (99.7 ± 0.01% [±SD] and 93.8 ± 0.02% [±SD], respectively; *SI Appendix*, Fig. S2 *G* and *H*). The fraction of other configurations (*L3-R3-R3-L3* and *R3-L3-L3-R3*) remained the same in *ΔmatP* and WT (*SI Appendix*, Fig. S2*L*), and *ΔmatP* daughter cells with flipped chromosome arms were initially born with the same orientation as in the mother cell (88.4 ± 2.8% [±SD]; *SI Appendix*, Fig. S2*F*). Overall, most *L3*-*R3*-*L3*-*R3* orientation flips to *R3*-*L3*-*R3*-*L3* (and vice versa) arose as a consequence of *L3*-*R3* to *R3*-*L3* flips (and vice versa) prior to locus duplication, followed by locus replication–segregation.

### Lagging Strand Segregation to the Cell Center, Marked by DnaN, Is Dependent on MukBEF.

Translational symmetry of sister chromosomes arises at least in part from the symmetric segregation of lagging strands toward midcell during DNA replication (and leading strands toward the cell poles), as shown using an elegant genetic system (21). Here, we sought directly to visualize the positioning of lagging strands in WT, *ΔmatP*, and *ΔmukB* cells.

**Fig. 3.**
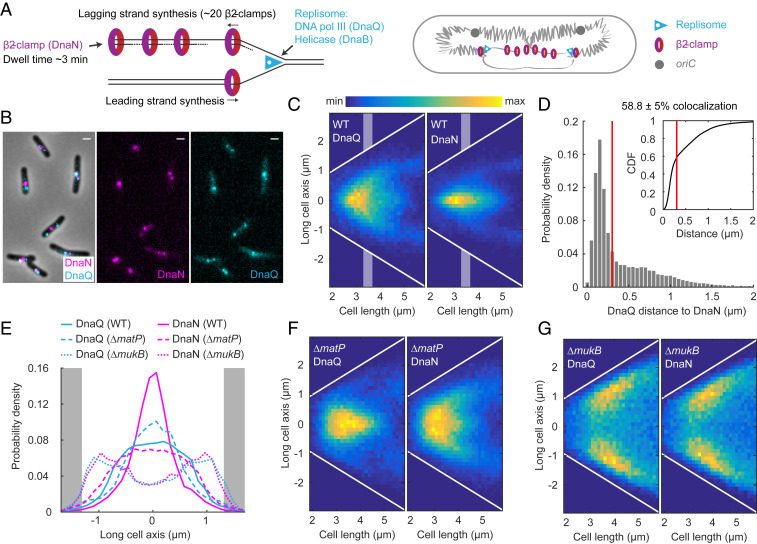
DnaN visualizes the lagging strands during replication. (*A*) Schematic of the accumulation of β_2_-clamps (DnaN) on the lagging strand during replication (33). The DNA polymerase ε-subunit (DnaQ) marks the location of the replisome. (*B*) Representative images of WT cells with fluorescently labeled DnaN and DnaQ. (Scale bars, 1 μm.) (*C*) DnaQ and DnaN localization in WT cells as a function of cell length (37,720 cells). White lines denote cell borders. Shaded areas denote intermediate cell lengths for localization data in *E*. (*D*) Distance from a DnaQ focus to the closest DnaN focus. DnaQ and DnaN colocalize in 58.8 ± 5% (±SD) of focus pairs (38,855 pairs), as defined by a distance threshold according to the diffraction limit (300 nm, red lines). Inset shows the same data as a cumulative distribution. The same data as in *C*. (*E*) DnaQ or DnaN localization with intermediate cell lengths (3.3 to 3.7 μm) in WT (DnaN 7,104 and DnaQ 8,006 spots), *ΔmatP* (DnaN 11,925 and DnaQ 8,025 spots), and *ΔmukB* (DnaN 5060, DnaQ 4205 spots) cells (see *C*, *F*, and *G*). Full width at half maximum of the distribution in WT: DnaN 0.67 ± 0.06 μm and DnaQ 1.67 ± 0.08 μm and in *ΔmatP*: DnaN 1.85 ± 0.04 μm and DnaQ 1.14 ± 0.14 μm (±SD). Gray areas denote cell poles. DnaQ and DnaN localization in *ΔmatP* cells (51,956 cells) (*F*) and in *ΔmukB* cells (22,902 cells) (*G*) as a function of cell length. White lines denote cell borders. Data are from three repeats in all analyses.

During replication, ∼40 DNA-bound β_2_-clamps, which ensure DNA polymerase III processivity, have a ∼3 min residence time on DNA before they are unloaded (33). The DNA-bound clamps are expected to accumulate largely on the lagging strand and its template because new clamps are loaded during synthesis of each Okazaki fragment (Fig. 3*A*). We reasoned that since β_2_-clamps could potentially cover >100 kb of newly replicated lagging strand DNA, they could serve as a marker to monitor lagging strand segregation. As a reference for the localization of replication forks, we imaged fluorescent DNA polymerase III ε-subunits (DnaQ) in the same cells. Indeed, while DnaQ foci were more spread toward cell poles, as previously described (34), DnaN foci localized closer to the cell center, consistent with the lagging strands being directed to midcell (Fig. 3 *B* and *C*). By measuring the distance from each DnaQ focus to the closest DnaN focus in each cell, we found that 41.2 ± 5% (± SD) of DnaQ foci do not colocalize (i.e., further apart than the diffraction limit dictates, ∼300 nm) with DnaN foci during replication (Fig. 3*D*). The differential location of DnaN and replication forks was confirmed by the measurement of the distances from replicative helicase (DnaB) foci to their closest DnaN focus (47.1 ± 6.1% [± SD] not colocalizing) (*SI Appendix*, Fig. S3 *A–C*). Since DnaN and DnaQ colocalize during early and late replication, when sister replisomes are necessarily close together, we also analyzed the localization patterns for cells that are in the middle of the replication cycle (Fig. 3*E*), when independently tracking replication forks are more frequently spatially separate. The pattern of DnaQ foci that did not colocalize with DnaN foci (*SI Appendix*, Fig. S3*F*) underlines the conclusion that spatially separate sister replisomes in opposite cell halves have a different cellular location from DnaN. Our results are consistent with the previous independent measurements of DnaQ and DnaN localization and the observation that DnaN foci of sister replisomes often do not spatially separate (34–36). Here, we provide direct evidence that the replisome and β_2_-clamps frequently do not colocalize during replication. Our visualization of the segregation of lagging strands during replication supports the previously shown symmetric segregation of leading strands toward the cell poles (21).

To analyze how MukBEF and MatP contribute to lagging strand segregation, we measured DnaN and DnaQ localization in *ΔmatP* and *ΔmukB* cells. The DnaN distribution in *ΔmatP* cells was broader than in WT cells (Fig. 3 *E* and *F*), indicative of spatially less precise lagging strand segregation but still directed toward cell centers, as predicted by the *L3*-*R3*-*L3*-*R3* organization. The DnaQ distribution in midcycle *ΔmatP* cells was more central than that of DnaN (50.8 ± 1.3% [±SD] colocalization with DnaN during replication; *SI Appendix*, Fig. S3*E*), most likely because of less separated chromosome arms, as shown by *L3* and *R3* markers (Fig. 2*F*). Both DnaQ and DnaN exhibited a broader distribution at shorter cell lengths (Fig. 3*F*), presumably because of a more random chromosome conformation (Fig. 2). *ΔmukB* cells showed a distribution of DnaN and DnaQ localizations toward cell poles, with almost identical patterns for both markers (Fig. 3*G* and 1 and 2 focus heatmaps in *SI Appendix*, Fig. S3 *H* and *I*). The results show that lagging strands and their templates cannot be directed to cell centers in a timely manner in the absence of MukBEF function, a result consistent with impaired *L3*-*R3* and *L3*-*R3*-*L3*-*R3* organization in *ΔmukB* cells (Fig. 2*G*). By measuring the distance from each DnaQ focus to the closest DnaN focus, we found that lagging strands did not leave the vicinity of the replisome during the DnaN dwell time on chromosomes of ∼3 min (78.4 ± 0.5% [±SD] colocalization; *SI Appendix*, Fig. S3*G*). We hypothesize that this is a consequence of delayed decatenation by TopoIV in the absence of MukBEF (37), since lagging strand templates can only be segregated from the leading strands once decatenation has occurred. Note that the generation times of WT, *ΔmatP*, and *ΔmukB* cells are comparable (18), with replication initiating and completing in the same cell cycle in most cells of all three strains (*SI Appendix*, Fig. S3*J*). This is in agreement with previous “runout” experiments (18), in which a fraction of *ΔmukB* populations having four chromosomes likely result from replication in the two-chromosome sister cells of an anucleate cell division.

We also examined the dynamin-like protein CrfC (aka YjdA), which has been proposed to bind β_2_-clamps and tether the nascent strands of sister chromosomes together (38). However, upon the deletion of *crfC*, we observed no changes to DnaN localization along the long cell axis (*SI Appendix*, Fig. S3 *K* and *L*) or any decrease in the frequency of the *L3*-*R3*-*L3*-*R3* configuration (*SI Appendix*, Fig. S3*M*). This result indicates that CrfC is not necessary for WT chromosome conformation and segregation.

### Ancestral DNA Strands Are Preferentially Retained at Older Cell Poles.

**Fig. 4.**
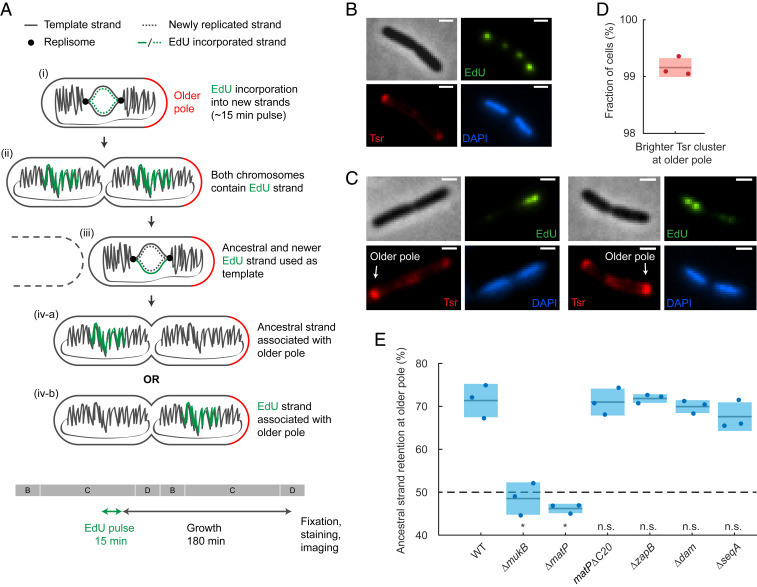
Visualization of ancestral DNA strand retention in *E. coli*. (*A*) Ancestral DNA strand propagation shown following an EdU pulse and the subsequent growth. After the second round of replication, only one of the chromosomes inherits the EdU label. Note that only a part of the chromosome is labeled with EdU. The 15-min EdU pulse and growth period are also shown relative to a schematic of cell-cycle stages (B, C and D periods; generation time ∼150 min). (*B*) Representative EdU^Alexa488^, Tsr^TMR^, and DAPI images of a cell at stage (ii) (see *A*) after the EdU pulse. Note that each chromosome has two EdU foci because pulse-labeled chromosome arms are separated. (Scale bars, 1 μm.) (*C*) Representative EdU^Alexa488^, Tsr^TMR^, and DAPI images after the complete pulse-chase protocol [stage (iv-a), see *A*]. The older pole is indicated with an arrow. (Scale bars, 1 μm.) (*D*) Accuracy of the older pole classification using Tsr prior to cell division. Shaded area denotes SD. Data are from 2,505 cells and three repeats. (*E*) Percentage of ancestral strand retained at the older pole in WT (988 cells), *ΔmukB* (427 cells), *ΔmatP* (1,050 cells), nondivisome-interacting *matPΔC20* mutant (1,617 cells), *ΔzapB* (969 cells), *Δdam* (717 cells), and *ΔseqA* (473 cells). The dashed line shows random retention. *P* value from two-proportion, two-tailed z-test was used to test if binomial distributions significantly differ from WT; indicated by n.s. (>0.01, nonsignificant) and * (<0.01, significant) (*P* values <10^−5^, <10^−5^, 0.51, 0.83, 0.26, and 0.03, respectively). Shaded areas denote SD from three repeats.

Previously, it has been hypothesized that a symmetrical segregation of lagging strands to the cell center leads to translational symmetry of sister chromosomes and, in consequence, the older template DNA strand (here referred as the ancestral strand since they are inherited from the grandmother generation or earlier) is not randomly segregated to daughter cells over subsequent generations but preferentially retained in the daughter with the older cell pole (discussed in ref. 20). Cell division generates two new cell poles at the division septum, while the other ends of the daughter cells are the older poles that were created in an earlier division. To address this theory directly, we developed a pulse-chase assay that allowed us to visualize the relative age of DNA strands between sister chromosomes and relate their position to the age of the pole without the need for cell synchronization or tracking (Fig. 4*A*).

The assay is comprised of the pulse labeling of newly replicated DNA and identifying the relative pole age by chemoreceptor accumulation at cell poles. The newly synthesized DNA was labeled by a 15 min EdU (5-ethynyl-2'-deoxyuridine) pulse, after which cells were washed and allowed to grow for 3 h (generation time ∼150 min). To avoid EdU-mediated growth defects, thymidine was added to the medium to outcompete EdU. We observed no detrimental effects on growth rate or cell size from the low concentration of EdU used in the pulse (*SI Appendix*, Fig. S4). After the growth period, most cells have completed the following round of replication, resulting in only one of the two sister chromosomes containing the EdU label (Fig. 4*A*). Cells were then fixed; EdU was visualized by click chemistry using Alexa 488 azide and nucleoids labeled by DAPI (Fig. 4*B*). As a result, in cells with completely replicated and segregated nucleoids (D-period), the chromosome with the newer template strand will be fluorescently labeled, while the one with the ancestral strand is not (Fig. 4*C* and *SI Appendix*, Fig. S4*D*).

To identify the older cell pole, we exploited the fact that the serine chemoreceptor, Tsr, accumulates approximately linearly with time at the cell poles (39). Hence, the older pole can be distinguished from the new pole by a higher quantity of fluorescently labeled Tsr. Because imaging the Tsr-GFP fusion used before (39) was incompatible with EdU staining, we used a functional HaloTag fusion of the endogenous *tsr* gene labeled with synthetic tetramethylrhodamine (TMR) dye. This allowed us to determine if the older strand chromosome was segregated toward the older or newer pole in each cell (Fig. 4*C*). In a control experiment, we confirmed that the intensity of Tsr-mYpet foci was higher at the older pole in 99.2 ± 0.5% (±SD) of cells (Fig. 4*D*).

We observed that 71.3 ± 3.9% (±SD) of WT cells contained EdU foci in the chromosome closer to the new pole (Fig. 4*E*). Because EdU was incorporated into the new template strand, this indicates that the ancestral strand is preferentially retained at the older pole. The result deviates significantly from random retention, in which the older pole would have a 50% chance of inheriting either strand (binomial two-tailed test *P* value < 10^−5^). We also compared the dispersion (SD) of our data to a binomial distribution with different sample sizes to estimate the reliability of our experiment (*SI Appendix*, Fig. S4*E*). We found excellent agreement, showing that our measurements are robust for the given sample size, with no additional noise sources, and increasing data sample size would give diminishing returns.

How is ancestral strand retention related to chromosome organization? To address this question, we tested the contributions of MukBEF and MatP to ancestral strand retention. Upon deletion of *mukB*, we observed a random segregation of the ancestral strand (48.5 ± 3.8% [±SD]; Fig. 4*E*), demonstrating that functional MukBEF is required for ancestral strand retention at older poles. Deletion of *matP* also abolished the preferential segregation of the ancestral strand (46.2 ± 1.1% [±SD]; Fig. 4*E*). While MatP has not been implicated in early chromosome segregation, when the segregation pattern(s) emerge, the influence of MatP on MukBEF action is crucial as it prevents chromosome arm flipping (Fig. 2*H*), which would disrupt the association of the ancestral strand with the older pole. MatP-*matS* also interacts with the divisome through ZapB, and this interaction has been proposed to partially anchor *ter* to the inner cell membrane (40). This interaction could plausibly contribute to the ancestral strand retention by anchoring the chromosome and thereby preventing chromosome rotation. However, upon replacing the native *matP* with a nondivisome-interacting *matPΔC20* mutant or deleting *zapB*, we did not observe any difference to WT with regard to ancestral strand retention (71.0 ± 3.1% and 71.8 ± 1.0%, respectively [±SD]; Fig. 4*E*). This confirms that the loss of ancestral strand retention in *ΔmatP* cells is related to the proposed chromosome rotation, measured by *L3* and *R3* flipping along the longitudinal cell axis over generations.

Finally, since MukBEF and MatP have coevolved with a group of proteins (including Dam and SeqA) that are related to Dam DNA methyltransferase activity (41), we tested the influence of these proteins on the retention of the ancestral strand. The delayed methylation of adenines in the sequence GATC transiently distinguishes the parental and newly synthesised strands after replication. Prior to Dam methylation, SeqA binds to hemimethylated GATC sites, negatively regulating replication initiation and possibly contributing to chromosome segregation (reviewed in ref. 42). Deletion of either *dam* or *seqA* did not influence ancestral strand retention at older poles (69.9 ± 1.5% and 67.6 ± 3.3%, respectively [±SD]; Fig. 4*E*), indicating that GATC methylation patterns do not affect the observed asymmetry and, consequently, overall *L*-*R* chromosome organization.

## Discussion

**Fig. 5.**
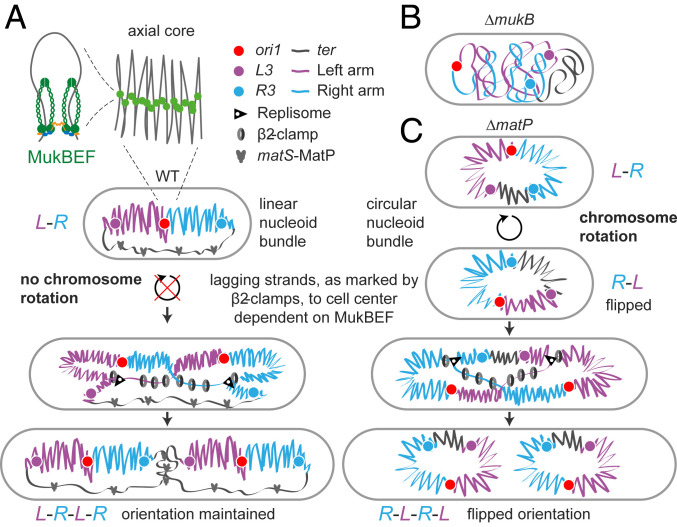
Chromosome organization and segregation by MukBEF and MatP. (*A*) MukBEF dimer of dimer complexes ubiquitously form DNA loops outside of *ter* from where MukBEF complexes are displaced by *matS*-MatP (17, 18, 51, 69). MukBEF action compacts the chromosome lengthwise forming a stiff linear chromosome bundle around an axial core (17, 47), which localizes chromosomal loci linearly along the long cell axis and directs chromosomes arms to opposite cell halves (*L*-*R*) (12, 32, 45). Our hypothesis proposes that the linear nucleoid bundle also restricts chromosome rotation, assayed by locus flipping, along the long cell axis. During replication and prior to division cells exhibit translational symmetric (*L*-*R*-*L*-*R*) segregation of sister chromosomes (12, 32), which is directed by the symmetric segregation of lagging strands and their templates during replication, dependent on MukBEF, as visualized by the accumulation of β_2_-clamps. Translational symmetric segregation also directs the inheritance of the older (immortal) template DNA strand, propagated from previous generations, to the cell forming at the old pole, dependent on MukBEF-MatP. (*B*) Absence of functional MukBEF reduces long-range chromosome folding (51), consequently increasing the effective contour length of chromosome. This causes mislocalization of chromosome loci and loss of *L*-*R* organization of the chromosome. (*C*) In the absence of MatP, MukBEF complexes are no longer displaced from *ter*, promoting lengthwise compaction of *ter* and the formation of a uniform, circular nucleoid bundle (17, 47). Thereby, chromosome arms cannot be efficiently directed to opposite cell halves, and nonreplicating chromosomes are free to rotate relative to the long cell axis, as indicated by the observed chromosome arm localizations. Any rotation between 90° and a complete 180° would flip the *L3*-*R3* locus orientation. Relative to FROS markers positions, replisome and β_2_-clamps localizations are derived from the data generated here, while axial core/nucleoid bundle architecture in WT and *ΔmatP* cells was characterized in refs. 17 and 47. The 1.42-Mb region between *L3* and *R3*, containing the 800-kb *ter* region and is depleted for MukBEF in WT cells (17, 18), is schematically displayed as a single black line, although it is compacted by other nucleoid-associated proteins.

Our results demonstrate how MukBEF directs the nucleoid organization and nonrandom segregation of sister chromosomes in *E. coli*. The rigorous analyses of genetic locus positioning in relation to the localization of MukBEF, replisomes, and newly replicated lagging strand, in a range of WT and mutant strains, provide insights into the molecular mechanisms underlying *E. coli* chromosome organization and segregation and complement previous studies that have quantified the nucleoid dynamics in mechanical terms (17, 31, 43, 44). The major observations are the following: 1) anucleate cells arise at the new pole in *ΔmukB* cells, and frequently in cells that have unsegregated *oriC* at the older pole cell half; 2) MukBEF and MatP have distinct roles in the generation and propagation of translationally symmetric chromosome organization over generations; 3) DNA-bound β_2_-processivity clamps, which mark lagging strands and localize to the cell center, dependent on MukBEF action and independent of replisome location; and 4) ancestral (immortal) DNA strands are preferentially retained in the sister cell with the older cell pole, dependent on MukBEF and MatP. We address how we interpret these observations below and present a model (Fig. 5) that integrates our conclusions and proposals with those of previous reports, thereby providing a conceptual foundation for understanding how nucleoid conformation and dynamics shape subcellular organization.

### *E. coli* Chromosome Organization.

The *E. coli* chromosome is organized into a nucleoid filament with chromosome loci positioned linearly along the longitudinal cell axis outside of the *ter* region (12, 32, 45, 46). Stiff nucleoid “bundles” that are radially confined by cell dimensions and exhibit a contour length of the scale of cell dimensions were characterized in live-imaging studies of *E. coli* (31). Bundles were also identified in cells with increased volume, which allowed the visualization of nonreplicating, toroidal chromosomes (47). Previous attempts to explain the precise chromosome loci positioning (e.g., by a randomly oriented polymer or transcription factor–mediated DNA loops) failed to provide the molecular requirements for maintaining chromosome conformation and orientation inside a cell (10, 43). We propose that the lengthwise compaction of the chromosome by a linear MukBEF axial core (17) can explain the formation of the nucleoid bundles (31) and the linear nature of chromosome loci positioning along the longitudinal cell axis outside of *ter* (Fig. 5). Linear MukBEF axial cores arise by the *matS*-MatP–mediated depletion of MukBEF from *ter*, which breaks the symmetry of otherwise circular chromosomes (17, 18). Continuous axial cores were observed in cells in which MukBEF occupancy on the chromosome was modestly increased (∼3.3-fold), while cells with WT cells exhibited more granular, but indistinguishable, MukBEF localization inside a cell (17). Theoretical studies have demonstrated that lengthwise compaction of the chromosome forms stiff bundles that promotes individualization of chromosome arms through excluded volume interactions and by the maximization of conformational entropy (48, 49). In the absence of MatP, MukBEF cannot be displaced from *ter*, and cells are unable to direct chromosome arms to opposite cell halves efficiently (Fig. 2 *F* and *G*). We propose that this is because the circular MukBEF axial cores of *ΔmatP* cells bring chromosome arms closer together than in WT cells (Fig. 5) (17). A less compacted and more “relaxed” *ter* region in WT cells might be required for efficient chromosome segregation during fast growth, since *ΔmatP* cells exhibit more frequent anucleate cell production than MatP^+^ cells (50).

The frequent *L3*-*R3* locus flipping in *ΔmatP* cells likely reflects global changes in the nucleoid, rather than local, locus-specific effects because genetic loci have predictable localization patterns in cells that recapitulate the physical and high-throughput chromosome conformation capture (Hi-C) contact maps of the chromosome (12, 32, 45, 46, 51). Furthermore, Hi-C analysis showed that deletion of MatP only affects chromosome organization in the *ter* region ∼300 kb away from *L3* and *R3* markers, and large excursions of chromosome loci were found to be rare outside replication–segregation of a specific locus (52). We therefore propose that the observed locus flipping can be explained by whole-chromosome rotation that displaces chromosomal loci along the longitudinal cell axis. Any intermediate value between 90° and a complete 180° rotation would flip the *L3*-*R3* locus orientation (Fig. 5*C*). In our model, in WT cells, a linear chromosome bundle [as opposed to uniform, circular chromosome bundle in *ΔmatP* cells (47)] restricts chromosome rotation, thereby explaining how *L3*-*R3*-*L3*-*R3* (or *R3*-*L3*-*R3*-*L3*) configuration can be stably propagated over generations without obvious membrane anchoring (Fig. 5). Other mechanism(s) may additionally contribute to the maintenance of chromosome orientation. A nondivisome-interacting MatP mutant displayed similar segregation behavior to WT, ruling out divisome tethering as a possible mechanism (see Fig. 4; ancestral strand retention would be lost if *L3*-*R3* flipping would occur like in *ΔmatP* cells). A chromosome membrane-tethering strategy is generally found in organisms in which MukBEF has been replaced by Smc-ScpAB complexes and which carry a *parABS* segregation system [e.g., through PopZ in *C. crescentus* (53), HubP in *Vibrio cholera* (54), and RacA/DivIVA in sporulating *B. subtilis* (55, 56)]. Membrane anchoring typically uses ParB bound to *oriC*-proximal *parS* sites as an intermediary. Intriguingly, some bacteria, such as *V. cholera* or *P. aeruginosa*, not only encode MukBEF/MksBEF but also specify a *parABS* system (9, 57). Whether organisms that encode MukBEF orthologs but not typical Smc-ScpAB complexes, and which lack ParABS systems, generally have life cycles that encompass overlapping replication cycles, similar to *E. coli*, remains to be determined.

In the absence of MukBEF, chromosome loci outside of the *ter* region were found to be generally more randomly localized (Fig. 2), in support of the hypothesis that MukBEF action positions the chromosome inside a cell through extensive intranucleoid interactions (51). The mislocalization of *oriC* toward older cell poles in *ΔmukB* cells may contribute to anucleate cell formation, since sister *oriC* need to move further apart than those in WT cells. An earlier analysis of locus positioning in *ΔmukB* cells led to the proposal that the impaired chromosome organization is frequently accompanied by the chromosome arms being aligned together along the long cell axis (16), an organization reminiscent of the situation in WT *C. crescentus* (3).

### Sister Chromosome Replication and Segregation.

A connection between translation symmetry of sister chromosome (*L*-*R*-*L*-*R*) and symmetrical segregation of leading/lagging strands has been previously proposed (20, 21). Consistent with this, we observed the accumulation of β_2_-clamps, present primarily on lagging strands, toward cell centers of replicating cells, when compared to both DNA polymerase III and helicase localization (Figs. 3 and 5). Differential positioning of the replisome and β_2_-clamps in WT cells also resolves the conundrum that emerged from studies that favored a model of a single-replication “factory” containing two replisomes at the cell center, based on clamp labeling (36). Our results support the model of independent tracking of the two often spatially separated replisomes in cells undergoing a single round of replication (34, 58), although segregation forces along with the reorganization of parental and newly replicated DNA leads to the frequent movement of sister replisomes toward the cell center.

A similar behavior was observed in *ΔmatP* cells, but in the absence of MukB, β_2_-clamps localized toward cell poles, coincident with replisomes. This shows that symmetric lagging strand segregation to the cell center determines the *L*-*R*-*L*-*R* segregation pattern of sister chromosomes, while a nearly random pattern of daughter chromosomes was observed in the absence of MukB. The presence of the *L*-*R*-*L*-*R* pattern in both WT and *ΔmatP* cells rules out chromosome orientation or chromosome arm separation as a requirement for establishing this pattern. We also refuted a previous hypothesis that a dynamin-like protein YjdA (aka CrfC) contributes to the symmetric lagging strand segregation by linking together the β_2_-clamp–loaded, nascent DNA strands (38). We hypothesize that MukBEF could plausibly differentiate between leading and lagging strands, leaving lagging strands less compacted (Fig. 5).

Our results also lead us to propose that the lifetime of individual, chromosome-associated clamps [estimated to be ∼3 min (33)] must be longer than the sister chromosome cohesion time for chromosomal regions outside of *oriC* and *ter* (estimated to be ∼14 min and ∼9 min, respectively) (18, 59). Precise measurements of cohesion times have been refractory to accurate experimental determination. Cohesion time between newly replicated sisters is at least partly determined by the time required for TopoIV to remove replicative catenanes (18, 37, 59), although tethering of *ter* to the divisome through MatP–ZapB interactions may also influence cohesion time in this region (60). MukBEF promotes TopoIV catalysis (18, 37). Therefore, delayed *oriC* segregation in *ΔmukB* cells, which was particularly evident in the relatively fast growing cells in the microfluidics experiments, could reflect impaired decatenation, since the decatenase TopoIV is no longer recruited by MukBEF to *oriC*-proximal regions. Indeed, modest overexpression of TopoIV led to a reduction in the cohesion time of newly replicated *oriC* from ∼14 min to ∼5 min in Muk^+^ cells (59). Delayed *ori* decatenation of *ΔmukB* cells may contribute to nonviability under fast growth conditions, while slow growth conditions allow sufficient time for chromosome decatenation and segregation in most cells. Nevertheless, the relative contributions of *oriC* mislocalization and delayed decatenation remain unknown.

### Ancestral Strand Retention at the Older Pole.

We have directly shown that the older template (“ancestral”) DNA strand is preferentially segregated to the older pole cell in *E. coli*. This nonrandom segregation is determined by the translational symmetry of the sister chromosomes (*L*-*R*-*L*-*R*), prior to cell division, and efficient maintenance of chromosome orientation over generations by MukBEF and MatP. However, as *E. coli* lacks the properties of cell differentiation, development, and regeneration of a multicellular organism, it is not clear why it has evolved a chromosome organization that nonrandomly segregates the ancestral strand to daughter cells. While *E. coli* cells can grow with a constant rate for hundreds of generations (30), the death rate was found to increase with replicative cell age, which was attributed to the growth-independent accumulation of protein damage (30). Increasing cellular maintenance processes through the general stress response reduced the death rate, while its absence increased it (61). Old pole cells have been shown to exhibit a diminished growth rate following the accumulation of cellular damage and misfolded protein aggregates (62, 63). The older pole also accumulates more membrane proteins (e.g., chemoreceptors and efflux pumps) than the new pole; this can significantly contribute to cell survival in challenging environments (39, 64). For example, the main multidrug efflux pump of *E. coli*, AcrAB-TolC, displays increased efflux activity in older pole cells compared to new pole cells, giving a growth advantage under subinhibitory antibiotic concentrations and possibly against other toxic compounds (64). AcrAB-TolC pump activity is also required for acquiring a resistance gene from mobile genetic elements in the presence of antibiotics (65). Finally, a common, epigenetic mechanism to regulate phase variation in bacteria involves the formation of DNA methylation patterns by proteins binding near a hemimethylated GATC site and blocking methylation (e.g., *pap* or *foo*, *clp*, and *pef* systems), which all encode pili (66). Preferential retention of the ancestral strand could potentially allow the old pole cell to maintain the previous methylated state. In the end, ancestral strand retention could simply be an evolutionary by-product of maintaining the *L*-*R* chromosome organization over replication–division cycles. Since ancestral strand retention occurs in only ∼70% of older-pole cells, this gives opportunities for selection in fluctuating or harmful environments, independent of whether older or newer pole cells thrive better.

## Materials and Methods

Detailed information of all experimental procedures is provided in *SI Appendix*. In brief, *E. coli* K12 AB1157 derived strains (*SI Appendix*, Table S1) were created using standard molecular biology and genetics techniques. Cells were grown in M9 0.2% glycerol minimal medium supplemented with five amino acids and thiamine at 30 °C, except for the microfluidics experiments in which cells were grown in M9 0.2% glucose supplemented with MEM amino acids and thiamine at 37 °C. For microscopy, cells were diluted 1,000-fold from an overnight culture, grown to an A_600_ of ∼0.1, and spotted on an M9 glycerol 1% agarose pad on a microscope slide or placed inside the microfluidics device, as in ref. 29. Inside the microfluidics device, cells were imaged every 5 min for >18 h. Agarose pad time lapses for chromosome arm flipping were imaged every 10 min for 3 h at 30 °C. Imaging was performed on a Nikon Ti-E microscope equipped with perfect focus system, 100× numerical aperture 1.4 oil objective, sCMOS camera (Hamamatsu Orca Flash 4), temperature chamber (Okolabs), and light-emitting diode excitation source (Lumencor SpectraX). For EdU experiments, cells were labeled with 10 μM EdU for 15 min, washed, introduced to fresh media containing 60 μg/mL thymidine, and allowed to grow for 3 h with shaking. Following this, cells were fixed, permeabilized, and a click chemistry reaction (Thermo Fisher Scientific, #C10337) was conducted using Alexa 488 azide. Finally, Tsr-HaloTag was labeled with TMR HaloTag ligand, as in ref. 67, and nucleoids were visualized by DAPI. All image analysis and cell tracking were performed using SuperSegger (68) in MATLAB (Mathworks). Further data analysis and statistics were also performed in MATLAB.

## Supplementary Material

Supplementary File

Supplementary File

Supplementary File

Supplementary File

## Data Availability

All study data are included in the article and/or supporting information.
