# Supplementary material for: Nonrandom segregation of sister chromosomes by Escherichia coli MukBEF
Source: Proc Natl Acad Sci U S A. 2021 Aug 12;118(33):e2022078118. doi: 10.1073/pnas.2022078118 (PMC8379921; doi:10.1073/pnas.2022078118)
Supplement: Supplementary File [file pnas.2022078118.sapp.pdf]

PNAS
www.pnas.org

Supplementary Information for
Non-random segregation of sister chromosomes by *Escherichia coli* MukBEF

Jarno Mäkelä, Stephan Uphoff, David J. Sherratt

Department of Biochemistry, University of Oxford, Oxford OX1 3QU, UK

Correspondence: jmakela@stanford.edu or david.sherratt@bioch.ox.ac.uk

This PDF file includes:

Supplementary text
Figures S1 to S4
Tables S1 to S2
Movies S1 to S3
SI References

Supplementary Information Text

Bacterial strains and growth conditions

Bacterial strains and primers are listed in Table S1 and S2, respectively. All strains were derivatives of *E. coli* K12 AB1157 (1). *kan*, *cat*, *gen*, and *hyg* refer to insertions conferring resistance to kanamycin (Km^r), chloramphenicol (Cm^r), gentamycin (Gm^r) and hygromycin B (Hyg^r), respectively. The insertions are flanked by Flp site-specific recombination sites (*frt*) that allow removal of the the resistance gene using Flp recombinase from plasmid pCP20 (2). *tsr-HaloTag-kan* and *tsr-mYpet-kan* were inserted into the native *tsr* chromosomal locus using λ-red recombination (2). The generated gene loci were transferred by phage P1 transduction to AB1157 yielding strains JM122 (*tsr-HaloTag-kan*) and JM133 (*tsr-mYpet-kan*). Deletion strains of JM122 were constructed by P1 transduction, first removing the *kan* resistance gene using Flp recombinase. *L3-R3* deletion strains were constructed from RRL66 using P1 transduction. The microfluidics strains (JM07 and JM09) were constructed from RRL189 by introducing *Δflhd::kan* and *ΔmukB::kan* by consecutive rounds of P1 transduction and Flp recombination. GFPmut2 cell marker was inserted at the *attTn7* site by a plasmid transformation as described in (3). The DnaQ and DnaN labeled strain (JM141) was constructed from RRL388 using P1 transduction from RRL36. Deletion strains of JM141 were constructed by P1 transduction, first removing the *kan* resistance gene using Flp recombinase. JM142 and JM143 were constructed by P1 transduction from JW4070. All genetic modifications were verified by PCR and/or sequencing and behavior in quantitative imaging. *mukB* deletions were verified by temperature-sensitivity in rich media, as described in (4).

Cells were grown in M9 minimal medium supplemented with 0.2% (v/v) glycerol, 2 μg ml^{-1} thiamine, and required amino acids (threonine, leucine, proline, histidine and arginine; 0.1 mg ml^{-1}) at 30 °C, except for the experiments in the microfluidics device (below). Cells grew with similar doubling times in WT, *ΔmatP* and *ΔmukB* strains (~150 min), as has been previously reported (4). For microscopy, cells were grown overnight, diluted 1000-fold and grown to an A_{600} of ~0.1. Cells were then pelleted, spotted onto an M9 glycerol 1% (w/v) agarose pad on a slide and covered by a coverslip. In mother machine microfluidics experiments, cells were first grown as above, and upon reaching A_{600} of ~0.1 were placed inside the microfluidics device, and the media running through the device was changed to M9 minimal medium supplemented with 0.2% (v/v) glucose, 2 μg ml^{-1} thiamine, MEM amino acids (Gibco, #11130-036), 0.1 mg ml^{-1} proline, and 0.85 mg ml^{-1} Pluronic F127 (Sigma-Aldrich, P2443), and microscope temperature was set to 37 °C. Under these faster growth conditions (generation time ~60 min for WT and *ΔmukB* cells), the cells are slightly fatter and so remain in the microfluidic device channels.

EdU pulse labeling

Cells grown until A_{600} of ~0.1 were labeled with 10 μM EdU (5-Ethynyl-2´-deoxyuridine, Thermofisher, C10337) for 15 min after which cells were washed, introduced to fresh media containing 60 μg/ml thymidine and allowed to grow for 3

h with shaking. Following this, cells were fixed with 4% PFA (v/v) for 30 min and permeabilized with 0.5% Triton X-100 (v/v) for 30 min. EdU click-chemistry reaction was conducted following the instructions (Thermofisher, #C10337) using Alexa 488 azide in a final volume of 50 μl for 30 min at room temperature, followed by washing. Cells were then labelled with TMR HaloTag ligand as in (5). Briefly, cells were incubated with 2 μM TMR ligand for 30 min and washed several times. Finally, nucleoids were labelled with 1 μg/ml DAPI for 15 min and washed, after which the cells were ready for imaging.

Epifluorescence microscopy

Fluorescence images were acquired on an inverted fluorescence microscope (Ti-E, Nikon) equipped with a perfect focus system, a 100× NA 1.4 oil immersion objective, a motorized stage, an sCMOS camera (Orca Flash 4, Hamamatsu), a temperature chamber (Okolabs), and an LED excitation source (Lumencor SpectraX). For non-time-lapse imaging, exposure times were 300 ms for TMR, Alexa 488, mCherry, mYpet; 150 ms for mCerulean; 100 ms for DAPI. Phase contrast images were collected for cell segmentation. Tsr time-lapse images were collected every 10 min for 3 h (19 frames), except Tsr-mYpet fluorescence signal was collected with an exposure time of 150 ms only for the last 2 h to minimize photobleaching. During microfluidics time-lapse experiments cells were imaged for more than 18 hours every 5 min (> 216 frames). Exposure times were 100 ms for GFP, 300 ms for mCherry, and 80 ms for CFP. Microscopy data was collected automatically from the sample area. Data statistics are shown in the description of each Figure.

Microfluidic devices

The microfluidic single-cell imaging device ("mother machine") was prepared as in (6). The device was designed using Autodesk AutoCAD software. The dimensions of the cell channels were 1.2 μm x 1.2 μm x 20 μm and the media flow channels were 100 μm x 25 μm. The structures were fabricated on a silicon wafer (Kavli Nanolab, Delft University) (7) and a negative polydimethylsiloxane (PDMS) mold was created from the silicon wafer using a 5:1 mixture of monomer and curing agent (Dow Corning Sylgard 184 Kit). After removing air bubbles using vacuum, the chip was cured at 65°C for 1.5 hours. The mold was treated with Trichloro(1H,1H,2H,2H perfluorooctyl)silane (Sigma) in vacuum overnight. The PDMS device was generated from the negative mold using a 10:1 mixture of monomer and curing agent and cured at 65°C for 1.5 hours. Media flow holes were punched through the device with 0.75 mm diameter. Cover slips were cleaned by sonication in acetone for 20 min, washing with dH2O, sonication in isopropanol for 20 min, and dried with nitrogen. The PDMS device was washed with isopropanol and dried with nitrogen. The device and a cover slip were bonded using air plasma (Plasma Etch PE-50) and placed in an oven at 95°C for 30 min. Cells were pipetted into the device and the device was centrifuged at 4000 rpm for 10 min to place cells into the channels. The media supplemented by Pluronic F127 was fed into the device through silicon tubing (Tygon ND 100-80 microbore, VWR) using a motorized infusion pump (New Era Pump Systems).

Initially, a high flow rate of 1.5 ml/h was applied to flush the excess cells and then lowered to 0.5 ml/h. After this, cells were allowed to grow for ~2 h before starting the time-lapse imaging.

Image analysis

Cell based information, including cell outlines, lineages, pole ages, per pixel fluorescence intensities, and fluorescent marker localization, was extracted using SuperSegger (8) in MATLAB (MathWorks). SuperSegger uses an image-curvature method to identify foci to avoid the identification of false positive foci due to background intensity from cytoplasmic fluorescence and uses a gaussian fit to find the subpixel resolution location of foci. Focus quality is determined by a combination of intensity and fitting parameters and bad quality foci were filtered out. A threshold value was confirmed by visual inspection and the same threshold was used for all compared data sets. SuperSegger uses a linking cost function computed from the spatial overlap between cell areas, the distance between the cell centroids, and the change in the cell areas between each successive frame to track cells in time-lapses. The linking cost information between frames was used to identify and remove cells that may have been incorrectly linked from further analysis (e.g., too large area change). Fluorescent channels were aligned prior to analysis.

Fluorescent marker localization

For heatmaps, single cell data from different cells in different imaging regions (and frames in time-lapses in Fig. 1) and experiments were combined and binned according to the fluorescent marker localization along the long cell axis and cell length. Each bin corresponds to the relative frequency of marker's long axis localizations as a function of cell length in a population, shown in a linear scale. As cell cycle events are correlated with cell size, this is a reasonable way to visualize the data. For *ori1, ter3, L3* and *R3* markers, binned marker density profiles with different cell lengths were normalized by the maximum value in each cell length bin, as the expectation is that a cell will always have at least a single focus. As the cell orientation is random relative to the pole age, cells were oriented to place *L3* more towards the negative pole than *R3* and, in the *ori1* data, *ter3* was oriented more towards the negative pole. To determine flipping frequency of *L3-R3-L3-R3* markers from time-lapse imaging, mother cells that contained *L3-R3-L3-R3* or *R3-L3-R3-L3* marker order were identified. Next, their daughter cells with *L3-R3-L3-R3* or *R3-L3-R3-L3* marker order were identified. The average angle (Fig. S2) between vectors pointing from the more polar *L3* to the more polar *R3* was calculated between a mother and daughter cells. If the angle exceeded 90°, the chromosome orientation was considered flipped. Number of *L3-R3-L3-R3* and *L3-R3* flipping events during cell cycle was measured by changes to the relative position of each marker in the long cell axis to other markers. To measure width of a unimodal distribution (Fig. 3E) and to avoid inaccuracy from binning the data, the data was fitted by a kernel distribution in MATLAB and full width at half maximum (FWHM) was calculated from the fitted distribution.

Mother machine analysis

From the lineage data, cells were classified as 'normal' growing cells, anucleate cells, mothers of anucleate cells and sisters of anucleate cells. Cells that disappeared early or did not have a tracked lineage were removed from the analysis. Anucleate cells were considered as cells that didn't divide, didn't elongate and lacked an *ori1* marker present, while its sister cell elongated and divided normally, and contained *ori1* marker(s). If neither of the sister cells grew and divided normally, cells were excluded from analysis. The older pole of the anucleate cell was traced back at least 2 generations to determine whether the anucleate cell formed on the older or newer pole of the mother cell. The cell size at birth (Fig. S1B) was determined at the first frame of each cell and the number of *ori1* foci prior to division (Fig. 1C) at the last frame of a cell. We did not observe significant decline in growth rate during time-lapse experiments (Fig. S1C).

EdU pulse labeling

A functional HaloTag fusion of the endogenous *tsr* gene was used in the EdU labeling, as click-chemistry reaction conditions are detrimental to conventional fluorescent proteins. To measure EdU association with the older pole, the following criteria were used to select cells from an asynchronous cell population (see Fig. S4). First, Otsu's thresholding (9) was used to segment nucleoid area(s) from the cellular background and only cells that have two separate, large-enough nucleoid areas were analyzed. Second, a cell must exhibit a clear difference in polar Tsr intensity. The center line of a cell was extracted to find coordinates of cell poles by fitting a cell mask to a second-order curve. The intersection of the cell mask border and the curve was used to define cell poles. Median Tsr intensity of the cell area was subtracted from all Tsr pixel intensities and a sum of 9 brightest pixels from each pole were used to quantify the pole intensity. To minimize effects of noise and discrete pixel size in segmentation, only cells with larger than 1.5-fold difference in polar Tsr intensity were analyzed. The pole that had a higher intensity of Tsr was designated as the older pole. Third, only one of the nucleoids have been labeled by EdU (EdU with short incorporation times appear as distinct foci, see Fig. 4 and Fig. S4). The foci below a fixed threshold for the score were discarded. The foci were mapped to the nucleoids by projecting coordinates on the center line of the cell. With these criteria, the processed microscopy data from SuperSegger was automatically analyzed to extract the result of EdU association with older cell pole. To avoid segmentation errors of the cell area, correct cell segmentation was visually inspected, and inaccurately segmented cells were removed.

Tsr time-lapse

The accuracy of Tsr-based identification of the old cell pole in our growth conditions was estimated by tracking cells with a functional mYpet fusion to the endogenous *tsr* gene over generations under a microscope. Only cells that both were born and divided during the time-lapse were analysed. Tsr intensity at each pole was calculated with same criteria as in the EdU experiment (see above). The accuracy of Tsr as the older pole marker was determined for each frame

separately by comparing results between Tsr intensity analysis and lineage tracking. The accuracy (Fig. 4D) was shown for the last frame prior to division to mimic the EdU experiment where only cells with segregated chromosomes were analyzed. We did not observe significant decline in growth rate during time-lapse experiments (Fig. S2B).

Supplementary Figures

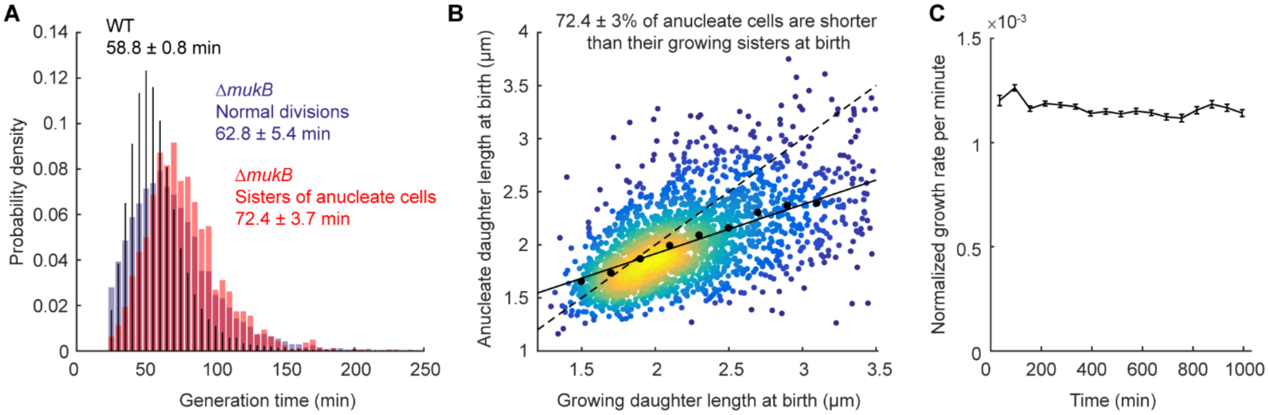

Figure S1. (**A**) Generation time (±SD) in WT cells (22470 cells), normally dividing *ΔmukB* cells (12103 cells) and sisters of anucleate cells in *ΔmukB* (1605 cells) from mother machine experiments. Two-sample t-test of mean generation time p-value 0.2176 between normally dividing and sisters of anucleate cells in *ΔmukB,* and p-value 0.5995 between normally dividing *ΔmukB* and WT cells. (**B**) Difference in cell length at birth between anucleate and growing sister cells at anucleate cell division (2266 cell pairs). Black dashed line indicates symmetric division and solid line shows a linear fit to the data. Black circles show binned mean. (**C**) Normalized growth in normally dividing cells during time-lapse imaging in mother machine (not including anucleate divisions, 12103 cells). The growth rate is defined as an increase in cell length divided by the cell length per minute. Error bars denote SEM. *ΔmukB* data are from 3 repeats and WT data from 2 repeats.

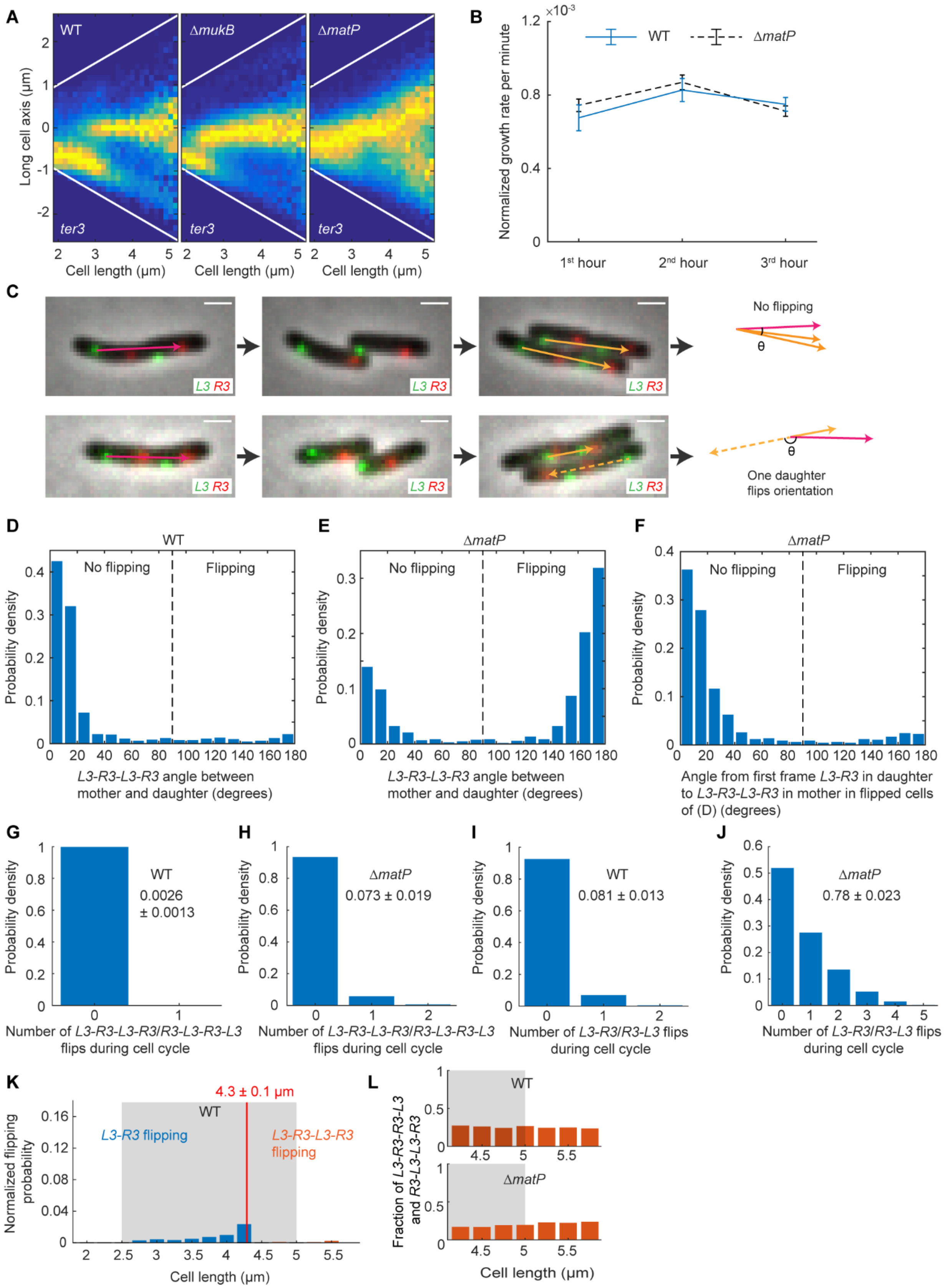
A
WT
ΔmukB
ΔmatP
ter3
Long cell axis (μm)
Cell length (μm)
B
×10-3
WT
ΔmatP
Normalized growth rate per minute
1st hour
2nd hour
3rd hour
C
L3 R3
No flipping
θ
One daughter flips orientation
D
WT
No flipping
Flipping
Probability density
L3-R3-L3-R3 angle between mother and daughter (degrees)
E
ΔmatP
F
Angle from first frame L3-R3 in daughter to L3-R3-L3-R3 in mother in flipped cells of (D) (degrees)
G
0.0026
± 0.0013
Number of L3-R3-L3-R3/R3-L3-R3-L3 flips during cell cycle
H
0.073 ± 0.019
I
0.081 ± 0.013
Number of L3-R3/R3-L3 flips during cell cycle
J
0.78 ± 0.023
K
4.3 ± 0.1 μm
L3-R3 flipping
L3-R3-L3-R3 flipping
Normalized flipping probability
Cell length (μm)
L
Fraction of L3-R3-R3-L3 and R3-L3-L3-R3

Figure S2. (**A**) *ter3* localization along the long cell axis in WT (26926 cells), Δ*mukB* (48770 cells) and Δ*matP* (45532 cells). Sample numbers with different cell lengths were normalized by the maximum value in each vertical bin. From same data as in Fig. 2D. *ter3* is oriented more towards the negative pole than *ori1*. (**B**) Normalized growth rate per minute during agarose pad time-lapses (Fig. 2H; WT 13911 cells, Δ*matP* 19317 cells). The growth rate is defined as an increase in cell length divided by the cell length per minute. Cells were imaged for 3 h every 10 min. Error bars denote SD between replicates. (**C**) Representative time-lapse images of WT (top) and *ΔmatP* (bottom) cells with *L3* and *R3* markers. (top) *L3-R3-L3-R3* orientation is maintained over a generation while (bottom) *L3-R3-L3-R3* orientation is flipped. From *L3-R3-L3-R3* cells, angle between vectors pointing from the more polar *L3* to the more polar *R3* is calculated between mother cell (red arrow) and daughter cells (orange arrows) and, if the angle exceeds 90°, the chromosome orientation is considered flipped (dashed arrow). Scale bars: 1 µm. Angle between mother and daughter cell *L3-R3-L3-R3* vectors in (**D**) WT (859 pairs) and (**E**) *ΔmatP* (1054 pairs) cells. (**F**) Angle between *L3-R3* vector in *ΔmatP* cells between the first frame of daughter cell and *L3-R3-L3-R3* vector in mother cell in flipped cells of (E). Number of *L3-R3-L3-R3* flipping to *R3-L3-R3-L3* (or vice versa) events during a cell cycle in (**G**) WT (2448 cells) and (**H**) *ΔmatP* cells (2434 cells). Mean number of flips and dispersion (±SD) between experiments are shown above each bar plot. Number of *L3-R3* flipping events to *R3-L3* (or vice versa) during a cell cycle in (**I**) WT (3059 cells) and (**J**) *ΔmatP* cells (4102 cells). Mean number of flips and dispersion (±SD) between experiments are shown above each bar plot. (**K**) Normalized probability of *L3-R3* flipping to *R3-L3* (or vice versa) (blue) and *L3-R3-L3-R3* flipping to *R3-L3-R3-L3* (or vice versa) (orange) as a function of cell length in WT (10362 cells). The flipping probability was normalized by the amount of data in each bin. Gray box indicates replication period as a function of cell size from Fig. 3. Red vertical line indicates cell length at locus duplication (±SD between experiments). (**L**) Fraction of *L3-R3-R3-L3 and R3-L3-L3-R3* configurations as a function of cell length in WT (4642 cells) and *ΔmatP* cells (5780 cells). Gray box indicates replication period as a function of cell size from Fig. 3. Data from 3 repeats in all analyses.

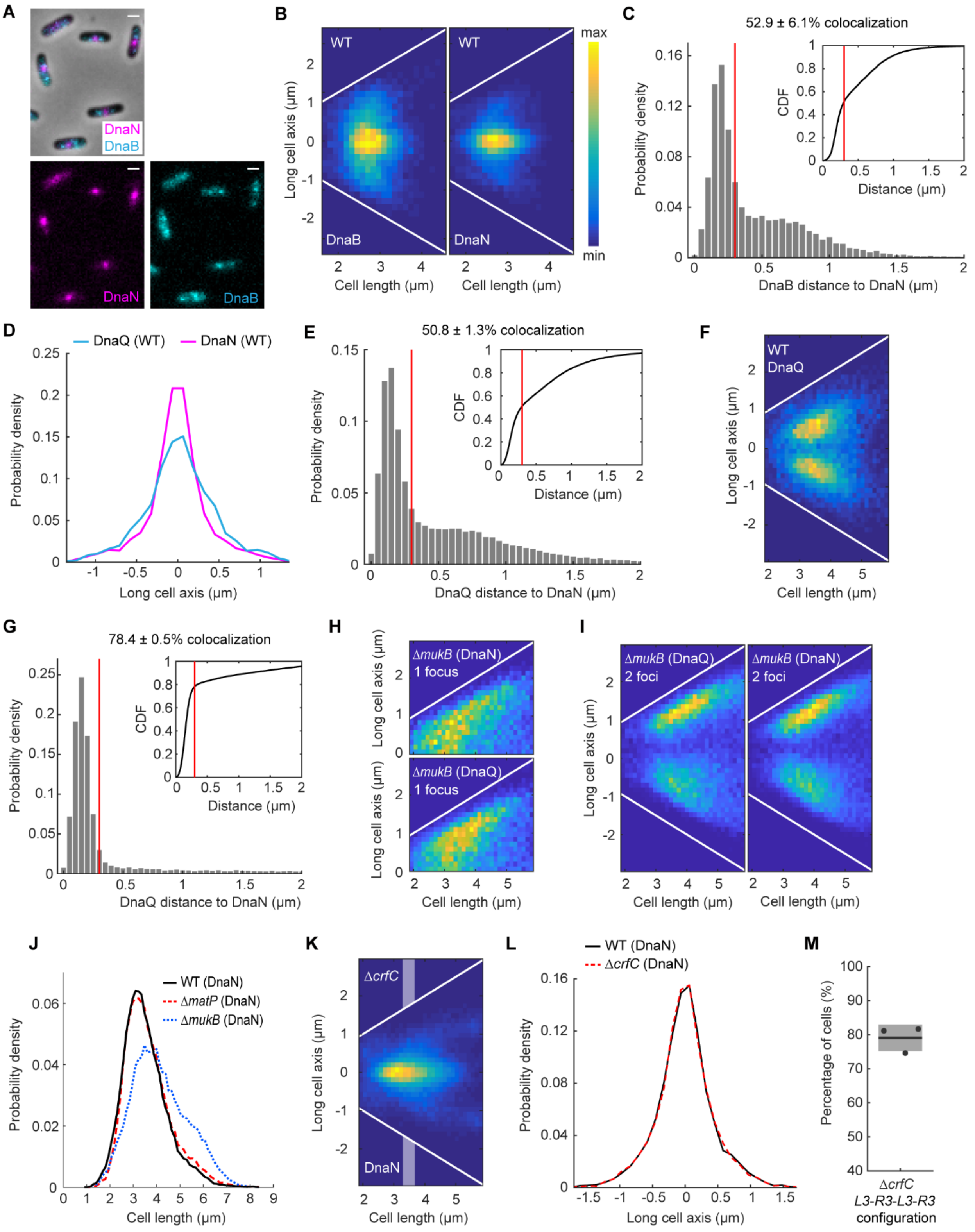

Figure S3. (**A**) Representative images of WT cells with labeled DnaN and DnaB. Scale bars: 1 µm. (**B**) DnaB and DnaN localization in WT cells as a function of cell length (16134 cells). White lines denote cell borders. (**C**) Distance from a

DnaB locus to the closest DnaN locus. DnaB and DnaN colocalize in 52.9 ± 6.1% (±SD) of pairs (11714 pairs) as defined by a threshold (red lines) below which two proteins colocalize (dictated by a diffraction limit of 300 nm). Inset shows the same data as a cumulative distribution. Same data as in (B). (**D**) DnaQ (4567 spots) or DnaN (5393 spots) localization with early replication cells in WT (cell lengths 2.5-2.9 μm) (same data as in Fig. 3C). (**E**) Distance from a DnaQ locus to the closest DnaN locus in *ΔmatP* cells. DnaQ and DnaN colocalize in 50.8 ± 1.3% (±SD) of pairs (46330 pairs). Inset shows the same data as a cumulative distribution. Same data as in (Fig. 3F). (**F**) DnaQ localization as a function of cell length in WT cells in which DnaQ foci are spatially separate from DnaN (16158 cells). Same data as in Fig. 2C and D. (**G**) Distance from a DnaQ locus to the closest DnaN locus in *ΔmukB* cells. DnaQ and DnaN colocalize in 78.4 ± 0.5% (±SD) of pairs (32603 pairs). Inset shows the same data as a cumulative distribution. Same data as in Fig. 3G. (**H**)-(**I**) DnaQ and DnaN localizations in *ΔmukB* cells as a function of cell length for (**H**) 1 focus cells and (**I**) 2 foci cells. Cells are flipped so that the more polar focus is always on the positive long cell axis. 8208 (36%) single DnaN focus cells, 11638 two DnaN foci cells (51%) and 2897 more than two DnaN foci cells (13%). 10108 (44%) single DnaQ focus cells, 10245 two DnaQ foci cells (45%) and 2390 more than two DnaQ foci cells (11%). Note that weaker DnaQ fluorescence signal leads to lower foci number estimate than DnaN. Same data as in Fig. 3G. (**J**) Normalized DnaN probability density as a function of cell length for WT, *ΔmatP* and *ΔmukB* cells. Same data as in Fig. 3C, F and G. (**K**) DnaN localization in *ΔcrfC* cells as a function of cell length (49955 cells). Shaded areas denote intermediate cell lengths for localization data in (L). White lines denote cell borders. (**L**) DnaN localization with intermediate cell lengths (3.3-3.7 μm) in WT (8006 spots) and *ΔcrfC* (10691). Data from (K) and Fig. 3E. (**M**) Percentage of *ΔcrfC* cells (8393 cells) with *L3-R3-L3-R3* (or *R3-L3-R3-L3*) configuration (versus *L3-R3-R3-L3* or *R3-L3-L3-R3*) in double *L3* and *R3* focus cells. Data from 3 repeats in all analyses.

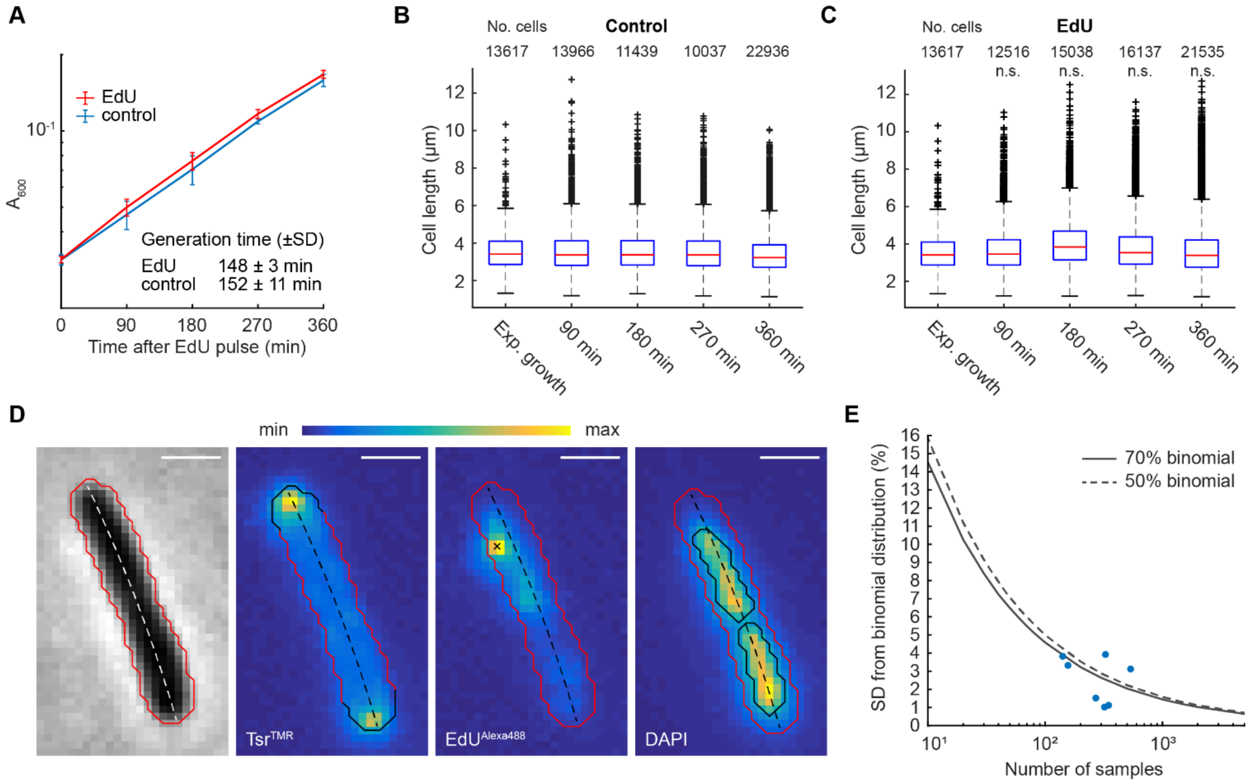

Figure S4. (**A**) Cell growth following a 15 min EdU pulse compared to no EdU. Cell length at different time intervals (**B**) without or (**C**) with EdU pulse. n.s. indicates two-sample t-test of mean cell length compared to control p-value > 0.01. (**D**) Image analysis of EdU experiment. A representative cell after EdU protocol showing Tsr^{TMR}, $EdU^{Alexa488}$ and DAPI labelling. Red line is the cell border and dashed line shows the center line of the cell. Black line in Tsr^{TMR} channel shows the pole areas from where the Tsr intensity is calculated. Black cross in $EdU^{Alexa488}$ channel indicates a detected EdU focus. Black lines in DAPI channel indicate segmented nucleoid areas. For more information see Methods. Scale bars: 1 µm (**E**) Accuracy of the retention measurement as function of sample size. Different sample sizes were drawn from a binomial distribution with 50% (dashed line) or 70% (solid line) success rate and SD was calculated between them (10^5 repeats for each value). The data from Fig. 4E are shown with dots. Data from 3 repeats in all analyses.

Supplementary Tables

Table S1. Strain list. *kan*, *cat*, *gen*, and *hyg* refer to insertions conferring resistance to kanamycin (Km^r), chloramphenicol (Cm^r), gentamycin (Gm^r) and hygromycin B (Hyg^r), respectively.

Strain	Relevant genotype	Source
AB1157	F^-, λ^-, rac^-, *thi-1, hisG4, Δ(gpt-proA)62, argE3, thr-1, leuB6, kdgK51, rfbD1, araC14, lacY1, galK2, xylA5, mtl-1, tsx-33, supE44(glnV44), rpsL31*(str^R), qsr'-0, mgl-51	Coli Genetic Stock Center (CGSC) #1157 (1)
AU2101	AB1157, *lacO*240 at *ori1* (3908) (*hyg*), *tetO*240 at *ter3* (1644) (*gen*), *ΔleuB::Plac-lacI-mCherry, ΔgalK::Plac-tetR-mCerulean, ΔmukB::kan*	(4)
JM07	AB1157, *lacO*240 at *ori1* (3908) (*hyg*), *tetO*240 at *ter3* (1644) (*gen*), *ΔleuB::Plac-lacI-mCherry, ΔgalK::Plac-tetR-mCerulean, attTn7::PRNA1-GFPmut2, Δflhd::kan*	This study
JM09	AB1157, *lacO*240 at *ori1* (3908) (*hyg*), *tetO*240 at *ter3* (1644) (*gen*), *ΔleuB::Plac-lacI-mCherry, ΔgalK::Plac-tetR-mCerulean, attTn7::PRNA1-GFPmut2, Δflhd, ΔmukB::kan*	This study
JM122	AB1157, *tsr::tsr-HaloTag-kan*	This study
JM127	AB1157, *tsr::tsr-HaloTag*, *ΔmatP::kan*	This study
JM128	AB1157, *tsr::tsr-HaloTag*, *ΔseqA::kan*	This study
JM130	AB1157, *tsr::tsr-HaloTag*, *Δdam::kan*	This study
JM131	AB1157, *tsr::tsr-HaloTag*, *ΔmukB::kan*	This study
JM133	AB1157, *tsr::tsr-mYpet-kan*	This study
JM135	AB1157, *lacO240 at L3 (2268) (hyg), tetO240 at R3 (852) (gen), ΔleuB::Plac-lacI-mCherry, ΔgalK::Plac-tetR-mCerulean, ΔmatP::kan*	This study
JM136	AB1157, *tsr::tsr-HaloTag*, *matP::matPΔC20-kan*	This study
JM137	AB1157, *tsr::tsr-HaloTag*, *ΔzapB::kan*	This study
JM140	AB1157, *lacO240 at L3 (2268) (hyg), tetO240 at R3 (852) (gen), ΔleuB::Plac-lacI-mCherry, ΔgalK::Plac-tetR-mCerulean, ΔmukB::kan*	This study
JM141	AB1157, *dnaN::mCherry-dnaN, dnaQ::dnaQ-mYpet-kan*	This study
JM142	AB1157, *dnaN::mCherry-dnaN, ΔyjdA::kan*	This study
JM143	AB1157, *lacO240 at L3 (2268) (hyg), tetO240 at R3 (852) (gen), ΔleuB::Plac-lacI-mCherry, ΔgalK::Plac-tetR-mCerulean, ΔyjdA::kan*	This study
JM150	AB1157, *dnaN::mCherry-dnaN, dnaQ::dnaQ-mYpet, ΔmatP::kan*	This study
JM152	AB1157, *dnaN::mCherry-dnaN, dnaQ::dnaQ-mYpet, ΔmukB::kan*	This study

JW4070	*ΔyjdA::kan*	Coli Genetic Stock Center (CGSC) #10929
RRL36	AB1157, *dnaQ::dnaQ-mYpet-kan*	(10)
RRL66	AB1157, *lacO240 at L3 (2268) (hyg), tetO240 at R3 (852) (gen), ΔleuB::Plac-lacI-mCherry, ΔgalK::Plac-tetR-mCerulean*	(10)
RRL189	AB1157, *lacO*240 at *ori1* (3908) (*hyg*), *tetO*240 at *ter3* (1644) (*gen*), Δ*leuB::Plac-lacI-mCherry*, Δ*galK::Plac-tetR-mCerulean*	(10)
RRL388	AB1157, *dnaN::mCherry-dnaN*	(11)
RRL396	AB1157, *dnaB::mYpet-dnaB, dnaN::kan-mCherry-dnaN*	(11)
SN192	AB1157, *lacO*240 at *ori1* (3908) (*hyg*), *tetO*240 at *ter3* (1644) (*gen*), *ΔleuB::Plac-lacI-mCherry, ΔgalK::Plac-tetR-mCerulean, mukB::mukB-mYpet*	(4)
SN302	AB1157, *lacO*240 at *ori1* (3908) (*hyg*), *tetO*240 at *ter3* (1644) (*gen*), *ΔleuB::Plac-lacI-mCherry, ΔgalK::Plac-tetR-mCerulean, mukB::mukB-mYpet, ΔmatP::cat*	(4)

Table S2. Primer list.

Name	Sequence	Construct
JMP48_Fw	CGCCGCGTAAAATGGCCGTGGCAGATAG CGAGGAGAACTGGGAAACATTTTCGGCT GGCTCCGCTGC	λ-red attachment of *HaloTag-kan* or *mYpet-kan* to *tsr* at the endogenous locus.
JMP49_Rv	AATCTCCTTATGCCCGATAACATTTTGCTT ATCGGGCATTTTCATGGCGATATGAATAT CCTCCTTAGTTCCTAT	

Supplementary Movies

Movie S1. An example time-lapse from Fig. 1. Mother machine microfluidics time-lapse of Δ*mukB* cells with *ori1* (red) and *ter3* (cyan) FROS markers, and constitutive expression of segmentation marker (green). 248 frames taken every 5 min.

Movie S2. An example time-lapse from Fig. 2H. Agarose time-lapse of WT cells with phase contrast, *L3* (red) and *R3* (cyan) FROS markers. 19 frames taken every 10 min.

Movie S3. An example time-lapse from Fig. 2H. Agarose time-lapse of Δ*matP* cells with phase contrast, *L3* (red) and *R3* (cyan) FROS markers. 19 frames taken every 10 min.

References

1. B. J. Bachmann, Derivation and Genotypes of Some Mutant Derivatives of *Escherichia coli* K-12. *Escherichia coli Salmonella typhimurium Cell. Mol. Biol.* **ASM**, 2460–2488 (1996).
2. K. A. Datsenko, B. L. Wanner, One-step inactivation of chromosomal genes in *Escherichia coli* K-12 using PCR products. *Proc Natl Acad Sci U S A* **97**, 6640– 6645 (2000).
3. G. J. McKenzie, N. L. Craig, Fast, easy and efficient: Site-specific insertion of transgenes into Enterobacterial chromosomes using Tn7 without need for selection of the insertion event. *BMC Microbiol.* **6**, 39 (2006).
4. S. Nolivos, *et al.*, MatP regulates the coordinated action of topoisomerase IV and MukBEF in chromosome segregation. *Nat. Commun.* **7**, 10466 (2016).
5. N. Banaz, J. Mäkelä, S. Uphoff, Choosing the right label for single-molecule tracking in live bacteria: Side-by-side comparison of photoactivatable fluorescent protein and Halo tag dyes. *J. Phys. D. Appl. Phys.* **52**, 064002 (2019).
6. S. Uphoff, Real-time dynamics of mutagenesis reveal the chronology of DNA repair and damage tolerance responses in single cells. *Proc. Natl. Acad. Sci. U. S. A.* **115**, E6516–E6525 (2018).
7. M. C. Moolman, Z. Huang, S. T. Krishnan, J. W. J. Kerssemakers, N. H. Dekker, Electron beam fabrication of a microfluidic device for studying submicron-scale bacteria. *J. Nanobiotechnology* **11**, 12 (2013).
8. S. Stylianidou, C. Brennan, S. B. Nissen, N. J. Kuwada, P. A. Wiggins, SuperSegger: robust image segmentation, analysis and lineage tracking of bacterial cells. *Mol. Microbiol.* **102**, 690–700 (2016).
9. N. Otsu, A threshold selection method from gray-level histograms. *IEEE Trans. Sys. Man. Cyber.* **9**, 62–66 (1979).
10. R. Reyes-Lamothe, C. Possoz, O. Danilova, D. J. Sherratt, Independent Positioning and Action of *Escherichia coli* Replisomes in Live Cells. *Cell* **133**, 90–102 (2008).
11. N. Soubry, A. Wang, R. Reyes-Lamothe, Replisome activity slowdown after exposure to ultraviolet light in *Escherichia coli*. *Proc. Natl. Acad. Sci. U. S. A.* **116**, 11747–11753 (2019).
